# A new logistic-type model for pricing European options

**DOI:** 10.1186/s40064-015-1563-9

**Published:** 2015-12-09

**Authors:** Jaime A. Londoño, Javier Sandoval

**Affiliations:** Departamento de Matemáticas y Estadística, Universidad Nacional de Colombia, Manizales, Colombia; Universidad Externado de Colombia, Bogotá, Colombia

**Keywords:** Volatility smile, Logistic model, European option

## Abstract

We propose a family of models for the evolution of the price process $S_t$ of a financial market. We model share price and volatility using a two-dimensional system of stochastic differential equations (SDEs) driven by a single Wiener process. We prove that this family of models is well defined and that each model from this family is free of arbitrage opportunities, and it is (state) complete. We use option prices written over the S&P500 from December 2007 to December 2008 to calibrate a model of the proposed family and compare the calibration results with results of the Heston Model for the same data set. The empirical results achieved in both models show similarities for periods of low volatility, but the model studied shows a better performance during the period of higher volatility.

## Background

We define a family of models where the evolution of the price process *S*(*t*) is given by the system of stochastic differential equations

$$\begin{aligned} dS_t&=(\sigma (\hat{S}_t)\theta -\delta +r) S_t\,dt+\sigma (\hat{S}_t) S_tdW_t\qquad S_0=s_0\\ d\hat{S}_t&=-\delta \hat{S}_t\,dt+(\sigma (\hat{S}_t)-\theta )\hat{S}_t\,d\,W_t\qquad \hat{S}_0=s_0. \end{aligned}$$

where $\sigma$ is a twice continuously differentiable function, and $\delta$, *r* and $\theta$ are constants for the dividend rate, the short interest rate and the market price of risk. We prove that as long as $\sigma (\cdot )$ satisfies a behavior defined by Eq. (11) below, the given system of differential equations describes a global solution (with non-explosion). The existence of a suitable solution is discussed since in the presented setup is not restricted to Lipschitz coefficients. We also prove that the market with stock whose price evolution is given by *S*(*t*) and short interest rate *r* is free of (state) arbitrage opportunities; in addition as long as $d \sigma (\cdot )/dx$ satisfies the non-singularity condition given by Eq. (12) the market defined above is (state) complete. The definitions of (state) completeness and (state) arbitrage opportunities are new and developed by the author (Londoño 2004). We also analyze the empirical behavior whenever $\sigma (x)=n_2(P-x)$, and $\theta =n_1/n_2$ where $n_1, n_2 ,P$ are constants; this family has a simple economic interpretation [see remarks make after Eq. (4)]. For the sake of clarity, we develop the theory for the particular model, and later we extend the results to the general family of models.

We analyze a particular model with better empirical properties than Heston’s model (Heston 1993). Moreover, this analyzed model for the evolution of all variables has a precise economic meaning, and the market model is a (state) complete market. Our model is simple, it is easy to calibrate, and it has reduced run times because it depends on very few parameters, and it captures most stylized facts observed in the market. Finally, the performance of our model is similar in terms of RMSE at regular times to Heston’s model. However, our model is better than the Heston’s Model in times with higher volatility and uncertainty (for example during the peak of the crisis of 2008). Also, the proposed model showed better dynamics of the volatility surface, showed evidence of autocorrelation in square log-returns, and there exists evidence of negative correlation between the volatility process and the level of prices. The model’s empirical properties are reviewed in "Some empirical facts".

The simplest model for equity prices is a geometric Brownian motion (see Black and Scholes 1973). Desirable properties of this model include market completeness along with the absence of arbitrage opportunities. Nevertheless, the Black-Scholes model has widely documented problems. There exists evidence of time, price and strike value affecting the volatility process of financial assets; those findings have been materialized on two concepts: *the volatility smile* and *the cluster effect*. Volatility clustering is the empirical observation that there appears to be high volatility and low volatility time periods. These effects have been empirically documented by Aït-Sahalia and Lo (1997), Jackwerth and Rubinstein (1996), Bollerslev et al. (1986), Derman (1999), Rebonato (2004), Derman (1999).

In order to overcome Black-Scholes’ shortcomings, research has extended this market model to allow for richer dynamics of financial asset prices.  Merton (1976), Derman and Kani (1998), Dupire (1994), Hobson and Rogers (1998), Hull and White (1987), Heston (1993), Hagan et al. (2002) proposed some of the extended models. Bakshi et al. (1997) reviewed some empirical performance of some of these alternative models.

In some cases those models violate market completeness; as consequence, unique prices under the absence of arbitrage will not be obtained (See Londoño 2004; Rebonato 2004; Broadie and Detemple 2004).

The family of models studied in this paper is an example of models where a pricing theory can be obtained using the results of Londoño (2008, 2004). In general, the theory developed in Londoño (2008) does not impose conditions on the eigenvalues of the volatility matrix. Traditional models usually require that the eigenvalues of the volatility matrix remain away from 0 (see Karatzas and Shreve 1998). The methodology developed in this paper gives sufficient conditions for the existence of non-explosive solutions and market completeness for the family of models proposed (see Remark 1). In general do not impose conditions on the eigenvalues of the volatility of the price process. The main results in this paper are complementary to results in  Londoño (2008) since it gives us concrete examples beyond standard models of financial markets.

In Londoño (2008), we developed a general theoretical framework for valuation and arbitrage. However, in order to provide models beyond the standard literature models, we needed to give conditions to guarantee the market completeness and the existence of non-explosive solutions for the stochastic differential equations than define the models. In this paper, we studied some of these conditions.

The contents of the paper are as follows. In "Definition and characterization of new logistic-type models" we develop a model for the evolution of the price of stocks and prove that an existence of non-explosive solutions for the stochastic differential equations that define the model. Moreover, we note that the model developed is free from (state) arbitrage opportunities. Also, we prove that the market determined by the model proposed is (state) complete, and we introduce the numerical methodology used to approximate asset prices as solutions to SDEs (stochastic differential equations). Also, we generalize the results obtained for a family of models that includes the model proposed. In "Model calibration" we present and compare the calibration results on the model presented with results obtained from the Heston model. In "Some empirical facts" we describe some empirical characteristics of the model.

## Definition and characterization of new logistic-type models

A first approximation to equilibrium theory is to assume short periods of time where the price of a stock is essentially constant, modulus arbitrage considerations. Namely if we consider an equilibrium price at the time 0 the short time evolution of the price of the stock should take into account non-arbitrage considerations and should account for changes in the interest rate and alike. If we believe that there is a short-term equilibrium price, and the observed market price is a noisy proxy the equilibrium price, then it is natural to have mean reversion in the volatility. However, any reversion should be on a changing equilibrium price (see “discussion” on Eq. (4) below).

From the above arguments, one of the possible one-dimensional stochastic extensions derived from the deterministic logistic equation under Londoño (2008) framework is:

1 $$\begin{aligned} dS_t=(r-\delta +n_1(P-\hat{S}_t))&S_tdt+n_2(P-\hat{S}_t)S_tdW_t\qquad S_0=s_0 \end{aligned}$$

where $r, \delta , P$, and $n_1,n_2\ne 0$ are real constants and $\hat{S}_t=S_tH_t$ is the financial asset price discounted by the state price density process,

2 $$\begin{aligned} dH_t=-H_t(r\,dt+\theta \,dW_t),\quad H_0=1 \end{aligned}$$

and *r* and $\delta$ are the constant interest rate and stock dividend yield respectively, $s_0$ is the price at the time 0 and the market price of risk is $\theta (t)=n_1/n_2$; this latter remark follows since

$$\begin{aligned} b_t+\delta -r=n_1(P-\hat{S}_t) \end{aligned}$$

$$\begin{aligned} \sigma _t=n_2(P-\hat{S}_t). \end{aligned}$$

where *b*(*t*), and $\sigma (t)$ are the return and volatility processes. We first notice that the system of equations defines a global (nonexplosive solution); see "Some basic properties". Throughout this paper we shall call the market defined by Eqs. (1), and (2) as the *linear model*.

We point out that the volatility can be 0 instantaneously, but as a consequence of Proposition 2 the set of time values where this occurs has measure 0.

It follows that

3 $$\begin{aligned} b_t-r+\delta =\sigma _t\theta . \end{aligned}$$

Therefore, there are not state tame arbitrage opportunities (see Londoño 2004). However, we notice that we allow singular volatilities. We have

4 $$\begin{aligned} \sigma _t=n_2H_t(PH_t^{-1}-S_t). \end{aligned}$$

We observe that the latter volatility is mean reverting against the process $PH_t^{-1}$. If we think that *P* is the short term equilibrium price at the time 0 and that *S* is the market price; then there is mean reversion towards the “short term equilibrium price” *P* at time *t*.

Finally as a consequence of Corollary 2 below the proposed model is complete. Market completeness together with an absence of arbitrage will provide consistent and unique prices for European contingent claims.

Models belonging to a logistic category have not been widely developed in modern research. Onyango (2007) proposed an extension of the logistic equations that govern population growth to model the behavior of an asset price for one-dimensional worlds. In Onyango (2007), asset prices are assumed to obey the following SDE (adapted to use the previous notation),

$$\begin{aligned} dS_t=n_1(P-S_t)S_tdt+n_2(P-S_t)S_tdW_t. \end{aligned}$$

The above framework is a direct extension of a deterministic logistic equation of the form,

$$\begin{aligned} dS=n_1(P-S)Sdt. \end{aligned}$$

However Onyango (2007) shows several sources of weakness. The most serious drawback on Onyango (2007) is the absence of a theoretical framework sustained by a complete and arbitrage-free market.

### Numerical approximation method

Due to the complexity of explicit solutions for some SDE systems, this work will use a numerical approximation procedure. Among all the methods in the current literature, we have chosen Wong-Zakai type approximations to be used due to its simplicity of implementation.

Wong and Zakai (1965) demonstrated that if the solution to an equation of the form,

5 $$\begin{aligned} dS_t= \tilde{b}(t,S_t)dt+\tilde{\sigma }(t,S_t)\circ dW_t,\qquad S_0=s_0 \end{aligned}$$

wants to be approximated using a partition $\{0=t_0<t_1<t_2\cdots <t_k=T\}$ of the time interval [0, *T*]; it is possible to accomplish it through the solution of the following ODE for each interval $[t_{i-1},t_i], i=1\cdots k$,

6 $$\begin{aligned}&\frac{dS^*}{dt}=\tilde{b}(t,S^*)+\tilde{\sigma }(t,S^*)(W_{t_i}-W_{t_{i-1}}) \nonumber \\&\text {with},\quad S^*_{t_i}=s_i,\qquad W_{t_i}-W_{t_{(i-1)}}\sim N(0,t_i-t_{i-1}). \end{aligned}$$

The last differential equation is in the sense of Stratonovich and $\tilde{b}(\cdot )$ and $\tilde{\sigma }(\cdot )$ are twice continuous differentiable functions on the spatial variable and continuously differentiable in the time variable. We define convergence of Wong-Zakai solution on an almost sure sense. See Wong and Zakai (1965) for more details.

The ODE system defined over the interval $\{0=t_0<t_1<t_2...<t_k=T\}$, which approximates the solution of the system of Eqs. 1 and 2 on each interval $[t_{i-1},t_i], i=1\cdots k$ is recursively solved using Matlab©ODE solvers and random number generators. The primary objective is to obtain approximations of *S*(*T*) to calculate European contingent claim prices with expiration *T*.

### Some basic properties

Next we develop the model proposed and proved that the system of proposed SDE’s defines a non-explosive solution. We should emphasize that this is not straightforward since the coefficients that define the SDE are not Lipchitz continuous.

Moreover, we prove that the model satisfies a condition that is given by Proposition 2 below. The latter condition implies that the model defined is (state) complete as a consequence of Londoño (2004, Theorem4.1). It should be emphasize that the results of this section are the core of this contribution and they can be summarized as mathematical results that give sufficient conditions to apply the theory of Londoño (2004).

We also point out that the results in this section generalize to any model defined by the system of SDE’s defined by Eq. (10) as long as the conditions given by Eqs. (11) and  (12) are met. Although this paper proposes a vast collection of models, we just review in detail a single model to illustrate properties and to give an overview of empirical properties that the model proposed has.

We first observe the *linear model* defined by Eqs. (1) and (2) is equivalent to

7 $$\begin{aligned} dS_t&=(r-\delta +n_1(P-\hat{S}_t)) S_t\,dt+n_2(P-\hat{S}_t)S_t\,dW_t\qquad S_0=s_0 \nonumber \\ d\hat{S}_t&=-\delta \hat{S}_t\,dt+(n_2P-\theta -n_2\hat{S}_t)\hat{S}_t\,d\,W_t\qquad \hat{S}_0=s_0. \end{aligned}$$

The solutions are equivalent in the sense that they produce the same solution for *S* and $\hat{S}$. Since the system of Eq. (7) is locally Lipchitz continuous there exist a unique local solution to the system of differential equations. In order to prove global existence, it is just sufficient to show that there is not an explosion in positive time. The latter consequence is a result of Proposition 1 below. First, using ItÃ$^{\prime }$’s rule we obtain the following lemma:

#### **Lemma 1**

*Assume the unique local solution of the differential equation*

$$\begin{aligned} d\hat{S}_t=\alpha (\hat{S}_t)\hat{S}_t\,dt+\beta (\hat{S}_t)\hat{S}_t\, dW_t\qquad \hat{S}_0=s_0>0 \end{aligned}$$

*for*$t\in [0,\tau )$*, where *$\tau$*is the explosion time for the differential equation on*$\mathbb {R}^+$*(the positive real numbers), and where *$\alpha (\cdot )$, $\beta (\cdot )$*are differentiable functions defined on*$\mathbb {R}^+$*. Then*$\hat{Y}_t=1/\hat{S}_t$, $t\in [0,\tau )$*is the maximal local solution of the differential equation*

8 $$\begin{aligned} d\hat{Y}_t=\left[ \beta ^2\left( \frac{1}{\hat{Y}_t}\right) -\alpha \left( \frac{1}{\hat{Y}_t}\right) \right] \hat{Y}_t\,dt-\beta \left( \frac{1}{\hat{Y}_t}\right) \hat{Y}_t\, dW_t\qquad \hat{Y}_0=1/s_0 \end{aligned}$$

As a consequence of the previous lemma, we obtain the following proposition:

#### **Proposition 1**

*There exist a unique non-explosive solution of the stochastic differential equation in*$\mathbb {R}^+=(0,\infty )$

9 $$\begin{aligned} d\hat{S}_t =\beta \hat{S}_t\,dt+(\alpha -\kappa \hat{S}_t)\hat{S}_t\,d\,W_t\qquad \hat{S}_0=s_0>0 \end{aligned}$$

*for any *$$\beta ,\alpha ,\kappa \in \mathbb {R}$$.

#### *Proof*

Since the coefficients of the previous SDE are differentiable, and, therefore, locally Lipschitz continuous, it follows that there exist a unique local maximal solution for the SDE for an random time interval $[0,\tau )$. Therefore, It is sufficient to prove that there is not explosion for any $T>0$ in $[0,\tau \wedge T)$ for the solution of the stochastic differential equation in $(0,\infty )$. Then, it is sufficient to prove that for any $T>0$

$$\begin{aligned} \limsup _{t\in [0,\tau \wedge T)} \hat{S}_t<\infty \qquad \text {and,}\qquad \liminf _{t\in [0,\tau \wedge T)} \hat{S}_t>0 \end{aligned}$$

almost everywhere. Assume $T>0$; we first prove that $\limsup _t \hat{S}_t<\infty$. For this, we notice that there exist a global solution (in the interval [0, *T*]) for the stochastic differential equation

$$\begin{aligned} dS^1_t=\left( \alpha -\kappa S^1_t\right) \left( \frac{\alpha }{2}-\kappa S^1_t\right) S^1_t\,dt +(\alpha -\kappa S^1_t) S^1_t\,dW_t\qquad \hat{S}_0=s_0 \end{aligned}$$

In fact there exist a closed form solution of a SDE whose drift and diffusion agrees with the coefficients of the previous differential equations outside a ball large enough. (see Kloeden and Platen 2011). Since for *x* large enough

$$\begin{aligned} \beta x\le (\alpha -\kappa x)(\alpha /2-\kappa x)x \end{aligned}$$

it follows, using a localization argument and stochastic inequalities [Karatzas and Shreve (1988, Proposition2.18)], that $\limsup _t \hat{S}_t<\infty$.

Finally, in order to prove that $\liminf _{t\in [0,\tau \wedge T)} \hat{S}_t>0$ for any $T>0$ it is sufficient to prove that $\limsup _{t\in [0,\tau \wedge T)} \hat{Y}(t)<\infty$, where $\hat{Y}_t=1/\hat{S}_t$. Since $\hat{Y}_t$ is the maximal local solution of the stochastic differential equation given by (8), $\hat{Y}_t$ satisfies

$$\begin{aligned} d\hat{Y}_t=\left( (\alpha ^2-\beta )\hat{Y}_t-2k\alpha +\frac{k^2}{\hat{Y}_t}\right) \,dt-\left( \alpha \hat{Y}_t-k\right) \, dW_t\qquad \hat{Y}_0=1/s_0. \end{aligned}$$

It follows from a localization argument and stochastic inequalities [Karatzas and Shreve (1988, Proposition2.18)] that any maximal solution of $\hat{Y}_t$ must be dominated from above by $\max (1,Y^u_t)$ where $Y^u_t$ the global solution of the following stochastic differential equation

$$\begin{aligned} dY^{u}_t=\left( (\alpha ^2-\beta )Y^u_t-2k\alpha +k^2\right) \,dt-\left( \alpha Y^u_t-k\right) \, dW_t, \qquad Y^u_0=1/s_0. \end{aligned}$$

We notice that the coefficients of the previous stochastic differential equation are Lipschitz continuous and therefore there exist a nonexplosive global solution in any interval [0, *T*]. It follows that $\sup _{t\in [0,\tau \wedge T]}\hat{Y}(t)<\infty$ as required where $\tau$ is the explosion time of $\hat{Y}_t$ exiting $(0,\infty )$. $\square$

#### **Corollary 1**

*There exist a unique global non-explosive solution to the system of stochastic differential equations of* (7).

#### *Proof*

By Proposition 1 there exists a unique non-explosive solution $\hat{S}_t$ to the second equation of the system of Eq. (7). It follows that there exist a closed form (non-explosive) solution of $S_t$ in terms of $\hat{S}_t$ [see for instance Karatzas and Shreve (1998, equation1.9)], using It Ã$^{\prime }$’s rule’s $\square$

Finally, we prove that the model proposed by the system of Eq. (7) is a (state) complete market. For this it is necessary the following proposition:

#### **Proposition 2**

*The unique non-explosive solution *$\hat{S}(t)$* of the stochastic differential equation  *(9)* has a density for any *$\beta ,\alpha ,\kappa \in \mathbb {R}\setminus \{0\}$* and any initial condition*$s_0>0$.

#### *Proof*

We notice that $\hat{S}(t)$ is also the solution in the sense of Stratonovich of the following SDE

$$\begin{aligned} d\hat{S}_t=\left( \beta -(\alpha -\kappa \hat{S}_t)(\frac{\alpha }{2}-\kappa \hat{S}_t)\right) \hat{S}_t\,dt +(\alpha -\kappa \hat{S}_t)\hat{S}_t\circ d\, W(t)\qquad \hat{S}_0=s_0>0. \end{aligned}$$

It follows by ItÃ$^{\prime }$’s rule that for any twice continuous differentiable function $f\in C^2$

$$\begin{aligned} df(\hat{S}_t)=A_0f(\hat{S}_t)\, dt+A_1f(\hat{S}_t)\circ dW_t \end{aligned}$$

where $A_0$ is the differential operator

$$\begin{aligned} A_0=\left( \beta -(\alpha -\kappa x)(\frac{\alpha }{2}-\kappa x)\right) x\frac{d}{dx} \end{aligned}$$

and $A_1$ is the differential operator

$$\begin{aligned} A_1=(\alpha -\kappa x)x\frac{d}{dx}. \end{aligned}$$

Then, the Lie bracket $[A_0,A_1]$ evaluated at $\alpha /\kappa$ is

$$\begin{aligned} \left[ A_0,A_1\right] _{\alpha /\kappa }=-\beta \frac{\alpha ^2}{\kappa }\frac{d}{dx} \end{aligned}$$

and, therefore, the result follows as a consequence of the Hörmander condition [ Hörmander (1967) and  Ikeda and Watanabe (1981)]. $\square$

#### **Corollary 2**

*The model given by the system of stochastic differential equations of *Eq. (7)* is free of (state) arbitrage opportunities, and it is (state) complete, where it is assumed that*$\delta ,n_1,n_2,P\in \mathbb {R}\setminus \{0\}$.

#### *Proof*

By construction, Eq. (3) holds and it follows by Londoño (2004, Theorem3.1) that the market is free of a state arbitrage opportunity. In order to prove that the proposed model is a (state) complete market we observe that the volatility for $S_t$ is $n_2(P-\hat{S}_t)$. It follows by Londoño (2004, Theorem4.1) that it is sufficient to prove that $\hat{S}(t)$ has a density, but this is the conclusion of Proposition 2. $\square$

#### *Remark 1*

If we assume a constant dynamic for the interest rate, the market price of risk and the dividend process (*r*, and $\theta$, $\delta$), then we can construct the new models in the following way. Let $\sigma (\hat{S})=f(\hat{S})$ where *f* is a function with two continuous derivatives. Define $b(\hat{S}_t)=f(\hat{S}_t)\theta -\delta +r$; if the system of stochastic differential equations

10 $$\begin{aligned} dS_t&=b(\hat{S}_t) S_tdt+\sigma (\hat{S}_t) S_tdW_t\qquad S_0=s_0\nonumber \\ d\hat{S}_t&=-\delta \hat{S}_t\,dt+(\sigma (\hat{S}_t)-\theta )\hat{S}_t\,d\,W_t\qquad \hat{S}_0=s_0. \end{aligned}$$

where $\hat{S}_t=H_tS_t$, is a system of no explosive differential equations. Londoño (2004, Theorem 3.1) implies that the system defines a market free of a state arbitrage opportunity. Using a similar argument to the one utilized in the Proposition  1 it can be shown that if

11 $$\begin{aligned} \limsup _{x\rightarrow \infty } \Big| \sigma \left( \frac{1}{x}\right) \Big| &<\infty \nonumber \\ \lim _{x\rightarrow \infty }\left\{ (\sigma (x)-\theta )^2+x\left( \sigma (x)-\theta \right) \frac{d \sigma }{d x}(x)\right\}&=\infty , \end{aligned}$$

then the system of equations is a non-explosive system. Using a similar argument to the one used in Proposition 2 it can be proved that as long as

12 $$\begin{aligned} d\sigma (x)/dx\ne \theta \qquad \text {for any }x>0\quad \text {with}\quad \sigma (x)=\theta \end{aligned}$$

then the market defined by the system of Eq. (10) is a (state) complete market. We can construct more models assuming a stochastic evolution of the interest rate and the market price of risk.

#### *Remark 2*

We notice that the standard theory of valuation and arbitrage is not well suited for the model proposed in this paper. The difficulty of the proposed model arises in the fact that the volatility of the price process *S*(*t*) given by Eq. (7) is allowed to take singular values. To overcome this difficulty we use the theory of arbitrage and valuation proposed in  Londoño (2004) and  Londoño (2008).

## Model calibration

Following standard procedures, the model is calibrated minimizing an error function. Shouten et al. (2004) considered absolute option price differences (AP). Another alternative is relative prices (RP) as in Mikhailov and Nögel (2003). In this paper, relative implied volatility (RV) differences (Shouten et al. (2004)) are used to implement the calibration procedure.

We define the error functions (average relative percentage error) as,

$$\begin{aligned} RV:=\sqrt{\sum _{i=1}^{n}\omega _i\biggl (\frac{IV^{mod}_i-IV^{mar}_i}{IV^{mar}_i}\biggr )^2}\\ \end{aligned}$$

where $IV^{mod}_i$ and $IV^{mar}_i$ is the implied volatility given by the model output and the market value of the corresponding option, and *n* is the number of options considered on any specified date and

13 $$\begin{aligned} \omega _i=\frac{1}{n^{mat}n_i^{str}} \end{aligned}$$

where $n^{mat}$ and $n_i^{str}$ are the number of maturities and the number of strikes with the same maturity as observation *i* respectively.

Since we do not know an analytical expression for the values of European contingent claims for the model proposed by the system of Eq. (7), we need to implement Montecarlo simulation techniques. In this document, the error function is minimized using a heuristic method of direct search. Specifically, we follow the Generalized Pattern Search (GPS) algorithm implemented by the function patternsearch on Matlab ©.

As benchmark model, we use the model proposed in  Heston (1993). We use Heston(93) model because it has analytical closed solutions (see  Carr and Madan 1999). Even though the Heston(93) model violates the market completeness assumption; market completeness is overcome by assuming a particular functional form for the volatility market price of risk.

### Volatility surface calibration results

#### Data

We recover the implied volatility surface from closing mid-prices of plain vanilla calls and puts written over the S&P500 every Tuesday from December 5th 2007 until December 3rd, 2008, using the Heston model (93) and the linear model. We filtered the database to leave options showing a daily trading volume greater than 1.000 transactions per day and expiration between 0.1 and 0.8 years. The latter will avoid illiquid option that will distort the calibration results.

The year 2008 represents a challenge to any model that intends to recover implied volatility from market option prices. Due to the liquidity crunch generated by the sub-prime crisis, market volatility peaked to one of the highest levels ever. For example, the VIX index reached 80 on October 27th and November 20th, 2008. This increase in volatility accompanied a decrease of 58.64 % in the S&P500 index.

On average, there are four different maturities and 54 options per day which fulfill the maturity and volume criteria. The calibration procedure assumed a fixed annual dividend yield of 1.89 % and 2007 average dividend yield according to Standard & Poor’s.

Because these models assume constant interest rates, they have been calibrated as follows. For the Heston(93) model, each option price was obtained using the corresponding spot interest rate. However, the linear model used the spot interest rate only for the first maturity. The linear model has been calibrated using a numerical approximation and asset price paths simulated for a particular maturity were also used to simulate asset prices on subsequent maturities and for these we used forward rates. In order to avoid discrepancies due to methodology implementation, calibration results using only closest maturity options are also reported. The spot interest rate curve was estimated from Treasury bill yields provided by the FED.

#### Results

**Fig. 1:**
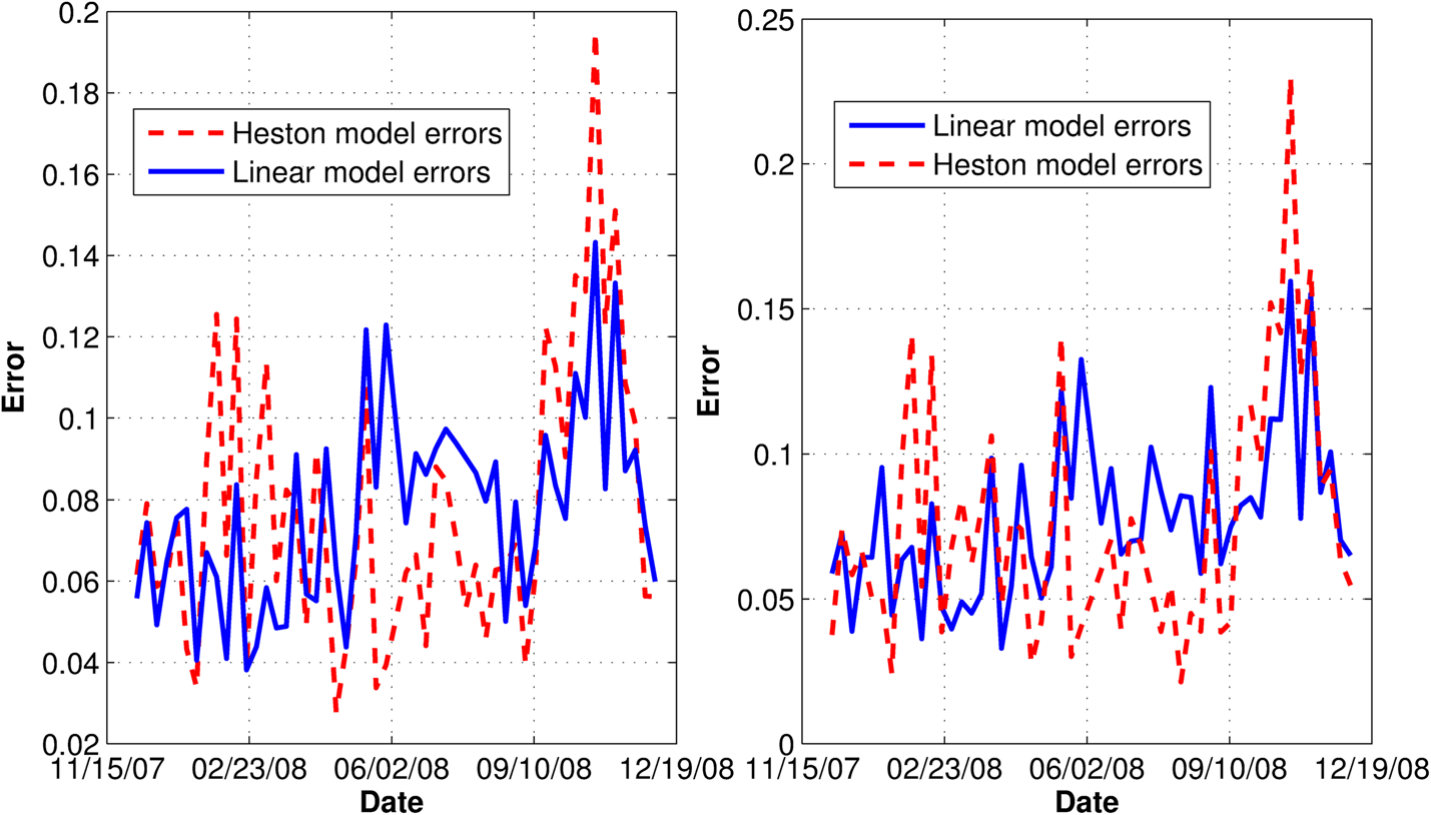
Error evolution for the calibration period using all data (*left*) and closest maturity options (*right*)

**Fig. 2:**
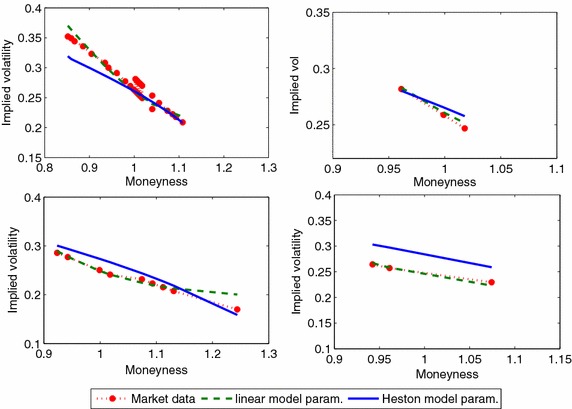
Implied volatility on February 6th, 2008 using market data, the model proposed on Eq. (7), and the Heston Model for different maturities. Mat: 0.12 (*upper*/*left*), 0.2 (*upper*/*right*), 0.37 (*bottom*/*left*) and 0.62 (*bottom*/*right*) years

We minimized the error function for 52 days based on relative implied volatility differences using all the option maturities. Figure 1 (left) depicts the error evolution for the calibration period. We provide Statistics on the error function are on the Table 1. Figure 2 shows the fit of models to market implied volatility on February 6th, 2008.

**Table 1:** Error statistics for the calibration procedure on the average relative percentage error for the linear model Eq. (7) and the Heston model

	Complete set		Closest maturity options	
	Linear model (%)	Heston model (%)	Linear model (%)	Heston model (%)
Mean	7.70	7.81	7.76	7.65
Stand. dev.	2.39	3.43	2.81	4.20

As observed in Table 1, the mean valued of the error function for the Heston model (93) and the linear model are similar. The evolution of the error function is similar even if we only used just the closest maturity options for calibration. Also, Table 1 also shows statistics of calibration results including closing maturity calls and puts (see Fig. 1 on the right side for evolution of errors using only options with the closest maturity).

Though the means of errors are similar as can be seen from Table 1, the standard deviation of the linear model error was lower than the one estimated using the Heston(93) model. Moreover, the linear model seemed to adjust better from September 17th to November 5th; that was the period with the highest volatility. The latter is a remarkable observation because there are as twice as many parameters in the Heston’s model(93) compared to the linear model.

**Fig. 3:**
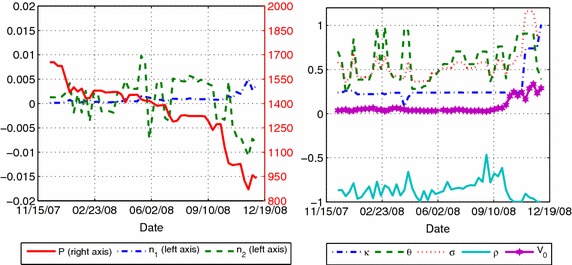
Evolution of model parameters given by Eq. (7) (*left*) and Heston(93) model (*right*). For the Heston Model the parameters are the ones defined by $dS(t)=\alpha S(t)dt+\sqrt{V(t)}S(t)dW(t)$, $dV(t)=\kappa (\Theta - V(t))dt+\sigma \sqrt{V(t)}dW^\sigma (t)$, and $E[dWdW^\sigma ]=\rho dt$

**Fig. 4:**
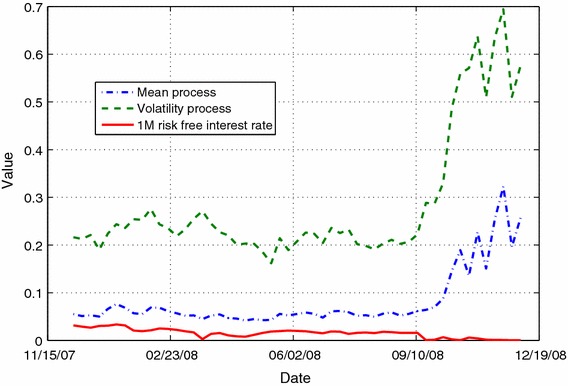
Volatility and Mean process of the model given by the system of Eq. (7) for the calibration period. The mean process has been estimated using the 1M risk-free interest rate

We show the evolution of the calibrated parameters for both models in Fig. 3. One particular observation is the behavior of the parameter *P* on the linear model. On average, *P* was located $7.04~\%$ above the initial index value ranging from $1.4~\%$ up to $14.70~\%$. This finding strengthens the idea of asset price level dependency of *P*. Also, Fig. 4 shows the volatility and drift of the price process *S*(*t*) of Eq. (7). As shown, the volatility and mean processes remain stable until September 17th, 2008. Afterward, they almost tripled. This increment is mainly explained by the disruption of the interbank credit market motivated by the bankruptcy or sell of several investment banks or commercial banks.

## Some empirical facts

**Fig. 5:**
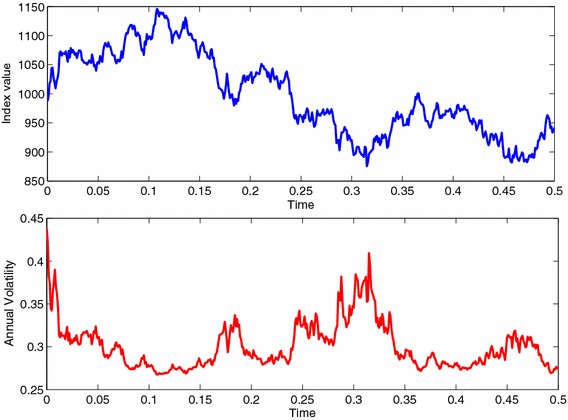
Price and volatility process for a randomly selected realization. $T=0.5, S(0)=987.5, P=1075.1, n_2=-0.003, r=1.57~\%, n_1=0.0013, \delta =1.89~\%$, and a partition of 500 points of the time interval

First, we assume a particular set of parameters for the linear model: $T=0.5, S(0)=987.5, P=1075.1, n_2=-0.003, r=1.57~\%, n_1=0.0013, \delta =1.89~\%.$ The dividend has been estimated using dividend yield reported by Standard & Poor’s. These parameters followed from calibration results in "Model calibration" over the S&P500 using relative volatility minimization on data from October 20th 2008. In Fig. 5 a random realization is chosen and its associated volatility process have been reported.

### Implied volatility smiles and parameter analysis.

Logistic features of the proposed model produce a high volatility regime when the discounted price is far from *P* and vice-versa. Results in Fig.5 also suggest that the model reproduces the world where the volatility process is negatively related to the stock price level. For this, let us denote as $\sigma _t=n_2(P-\hat{S}_t)$ the volatility of the price process given by Eq. 7, and $Y_t=\log ( S_t)$ the logarithmic of the price process. it follows by Itô’s calculus that $(Y_t,\sigma _t)$ satisfies the stochastic differential equation

14 $$\begin{aligned} dY_t&=(r-\delta +\theta \sigma _t-(1/2)\sigma _t^2)\, dt+\sigma _t\,dW_t\qquad Y_0=y_0 \nonumber \\ d\sigma _t&=n_2\delta \left( P-\frac{\sigma _t}{n_2}\right) \,dt-n_2(\sigma _t-\theta )\left( P-\frac{\sigma _t}{n_2}\right) \,dW_t\qquad \sigma _0=n_2(P-e^{y_0}). \end{aligned}$$

Therefore, the correlation between instantaneos increments is

$$\begin{aligned} Corr_t(dY_t,d\sigma _t)=sgn(\sigma _t(\sigma _t-\theta )(\sigma _t-n_2P))=-sgn(\sigma _t(\sigma _t-\theta )) \end{aligned}$$

on those points where $\sigma _t\ne 0$, and $(\sigma _t-\theta )\ne 0$, where $sgn(\cdot )$ denotes the sign of the given expression. The latter identity follows since $\hat{S}_t=P-\sigma _t/n_2>0$. It follows that as long as $n_2<0$ and $n_1>0$, then $Corr_t(dY_t,d\sigma _t)=-1$.

The leverage effect is a standard effect found in real data, and it is usually explained due to leverage reasons. In a leveraged company, if the stock price decreases, the company Debt/Equity ratio will increase though the level of debt is unchanged. An increasing leverage causes a high volatility level. This economic interpretation was developed by  Black (1976) and  Christie (1982). See for example Campbell and Hentschel (1992) for more details on the leverage effect evidence, and  Roman et al. (2008) for other models on this issue. Some recent developments in leverage estimation and problems arising with estimation of the leverage effect using high-frequency data are discussed by Aït-Sahalia et al. (2013).

### Floating smile and volatility surface

**Fig. 6:**
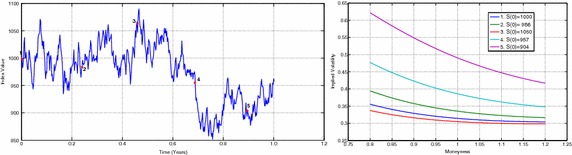
Evolution of implied volatility of the linear model for a fixed maturity time interval of $T=1$ year. Left: Simulated path of underlying price using $P = 1075.1, n_2 = 0.005, r = 1.57~\%, n_1 = 0.0013$ and $\delta = 1.89~\%$. Right: Implied volatility curves for 1-year maturity calculated using the linear model at 5 selected underlying prices

As discussed before a desirable model feature is the ability to reproduce floating smiles. Figure 6 (left) shows a simulated path of underlying price using $P = 1075.1, n_2 = 0.005, r = 1.57~\%, n_1 = 0.0013$ and $\delta = 1.89~\%$ and (right) several implied volatility curves calculated using the linear model at 5 selected underlying prices.

**Fig. 7:**
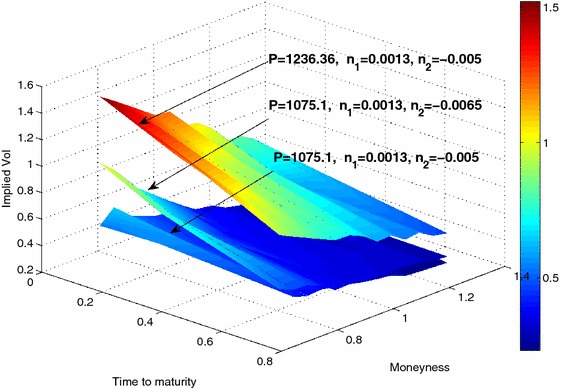
Volatility surface derived from the model given by the system of Eq.  (7) for times $\tau _1=0.1,\tau _2=0.3, \tau _3=0.7$ years

As we can see in Fig. 7, the volatility surfaces can recover implied volatility steepness, especially on short maturity options. We observe this particular feature on volatility surfaces extracted from option market data over the S&P500 from December 5th, 2007 to December 3rd, 2008.

#### Cluster effect in the volatility process

**Fig. 8:**
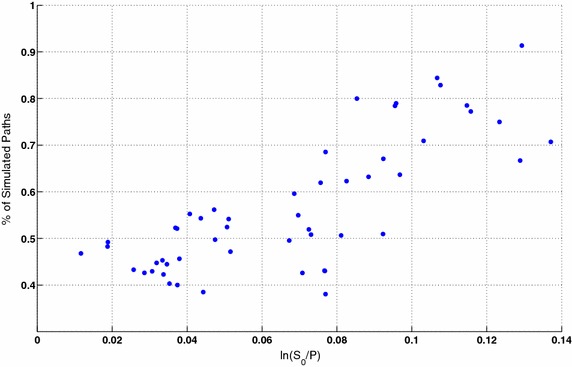
Percentage of simulated price process that showed significant autocorrelations different from zero using a $95~\%$ confidence interval with a fixed number of lags $L=20$. Each point represents a different set of parameters for the linear model calibrated from the $S \& P500$ option prices at every Tuesday of each of the 52 weeks starting at Dec-5-2007

One of the common features found in price processes is volatility clustering. Volatility clustering is the empirical observation that there appears to be high volatility and low volatility time periods. A Ljung-Box Q test was done over squared returns on the simulated prices using the parameters obtained from calibration of the linear model from $S \& P500$ option prices each Tuesday of each of the 52 weeks starting at Dec-5-2007. The null hypothesis was that the series of squared returns exhibits no auto-correlation for a fixed number of lags $L=20$. The alternative hypothesis is that some auto-correlation coefficient $\rho (k), k = 1, \ldots , L$, is nonzero. The test statistic is

$$\begin{aligned} Q=n(n+2)\sum _{k=1}^L\frac{\rho ^2(k)}{n-k} \end{aligned}$$

where *n* is the sample size, *L* is the number of autocorrelation lags, and $\rho (k)$ is the sample autocorrelation at lag *k*. Under the null hypothesis, the asymptotic distribution of *Q* is chi-square with *L* degrees of freedom. The test was done 10.000 times with $\alpha =5~\%$. We present results of the simulations on Fig. 8.

Simulations suggest that there exist a relation between correlation over squared returns and $P/S_0$, where *P* is the parameter that represent the "equilibrium price" and $S_0$ is the current price. For a discussion on the "equilibrium price" *P* see Eq. (4).

**Fig. 9:**
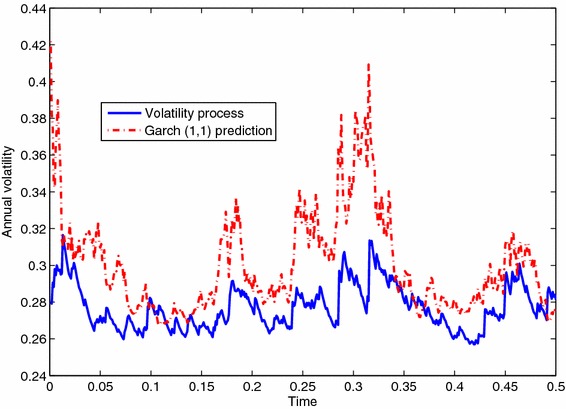
Volatility process and corresponding one-period ahead Garch(1,1) forecasting for a randomly selected index value path from the model given by the system of Eq. (7)

Finally as a consequence of persistence, a standard procedure for the GARCH family can be used to implement volatility forecasting. Figure 9 shows a simulated volatility process and the corresponding calibrated GARCH(1,1) fitted over the same selected stock price realization obtained from the linear model calibrated on October 20, 2008.

## Conclusions

We studied a class of models for the evolution of price process of a financial market that are complete. On this giving class, we can provide the existence of the stochastic differential equations that define the process as well as completeness. We also study a particular model from the proposed class, and the model proved to be simple and to behave better that Heston Model concerning Calibration for data on the S&P500 on 2008. Future lines of research include closed form solutions, calibration results for other models of the given class.
